# Mitochondrial phosphoenolpyruvate carboxykinase (PEPCK-M) regulates the cell metabolism of pancreatic neuroendocrine tumors (pNET) and de-sensitizes pNET to mTOR inhibitors

**DOI:** 10.18632/oncotarget.21665

**Published:** 2017-10-09

**Authors:** Pei-Yi Chu, Shih Sheng Jiang, Yan-Shen Shan, Wen-Chun Hung, Ming-Huang Chen, Hui-You Lin, Yu-Lin Chen, Hui-Jen Tsai, Li-Tzong Chen

**Affiliations:** ^1^ National Institute of Cancer Research, National Health Research Institutes, Tainan, Taiwan; ^2^ Department of Pathology, Show Chwan Memorial Hospital, Changhua, Taiwan; ^3^ School of Medicine, College of Medicine, Fu Jen Catholic University, New Taipei City, Taiwan; ^4^ Institute of Clinical Medicine, National Cheng Kung University, Tainan, Taiwan; ^5^ Department of Surgery, National Cheng Kung University Hospital, Tainan, Taiwan; ^6^ Department of Medicine, Taipei Veterans General Hospital, School of Medicine, National Yang-Ming University, Taipei, Taiwan; ^7^ Department of Internal Medicine, National Cheng Kung University Hospital, Tainan, Taiwan; ^8^ Department of Internal Medicine, Kaohsiung Medical University Hospital, Kaohsiung, Taiwan; ^9^ Institute of Molecular Medicine, National Cheng Kung University, Tainan, Taiwan

**Keywords:** pancreatic neuroendocrine tumor, PEPCK-M, mTOR, glycolysis, mitochondrial OXPHOS

## Abstract

mTOR pathway activation and hypervascularity have been identified as important characteristics of pancreatic neuroendocrine tumors (pNETs). Agents targeting angiogenesis and mTOR, such as sunitinib and everolimus (RAD001), have been shown to result in progression-free survival of approximately 11 months in patients with advanced pNETs. Novel treatment is needed to extend survival. Mitochondrial phosphoenolpyruvate carboxykinase (PEPCK-M), which is encoded by *PCK2*, catalyzes the conversion of oxaloacetate to phosphoenolpyruvate. PEPCK-M has been demonstrated to potentiate cytoplasmic phosphoenolpyruvate carboxykinase (PEPCK-C)-mediated gluconeogenesis and to play a critical role in the survival program initiated upon stress during metabolism in cancer cells. Elevated expression of *PCK2* has been found in various tumors according to the results of The Cancer Genome Atlas project. However, the role of PEPCK-M aberration in cancers is not well understood. In the current study, we observed that 12 of 21 (57%) pNET patients had high expression of PEPCK-M in the tumors, whereas the normal islet cells had weak expression of PEPCK-M. Knockdown of *PCK2* inhibited the proliferation of pNET cells and enhanced the sensitivity of pNET cells to mTOR inhibitors. Knockdown of *PCK2* promoted glycolysis but reduced mitochondrial oxidative phosphorylation in pNET cells. The combination of mTOR inhibitors and an anti-glycolysis agent, 2-DG, synergistically or additively inhibited the proliferation of pNET cells, particularly for the cells with high expression of PEPCK-M. Therefore, targeting PEPCK-M or glycolysis combined with inhibiting mTOR is a potential therapeutic approach for the treatment of pNETs.

## INTRODUCTION

Neuroendocrine tumors (NETs) are neoplasms that originate from neuroendocrine cells in different sites throughout the body. NETs were first described as carcinoids by Oberndofer in 1907 [1]. The incidence of NETs has increased in recent years, as reported in cancer registry databases in the US, Norway and Taiwan [2-4]. Pancreatic NET (pNET) is the fourth most common site of NETs in the US and Taiwan [2-4]. Although pNETs can be cured by surgery at an early stage, most pNET patients are diagnosed at an advanced stage. Everolimus, a mechanistic target of rapamycin (mTOR) inhibitor, and sunitinib, a multi-targeted tyrosine kinase inhibitor, have been demonstrated to extend the progression-free survival of patients with advanced pNETs; these drugs target mTOR and anti-angiogenesis based on the hypervascularity and activation of the mTOR pathway in pNETs [5-10]. However, even with these treatments, more than half of the patients develop disease progression after 11 months. Because the pathogenesis of pNETs is still not well understood, it is crucial to find new therapeutic agents for advanced pNETs.

Most cancer cells predominantly produce energy by anerobic glycolysis rather than by oxidative phosphorylation (OXPHOS), which has been termed the Warburg effect [11]. Moreover, the nutritional status of the tumor microenvironment may not be the same as that of normal tissues. Hirayama et al. [12] observed significantly lower glucose levels in tumor tissues than in normal tissues of colon and gastric cancers, whereas the levels of tricarboxylic acid (TCA) cycle metabolites such as succinate, fumarate, and malate were significantly higher in these tumor samples. Moreover, the accumulation of various amino acids was noted in tumor tissues. This result suggests that tumor cells also rely on aerobic respiration via the TCA cycle [12].

Phosphoenolpyruvate carboxykinase (PEPCK) catalyzes the conversion of oxaloacetate to phosphoenolpyruvate (PEP). There are two isozymes distributed in the cytosol (PEPCK-C) and mitochondria (PEPCK-M), which are encoded by *PCK1* and *PCK2*, respectively [13, 14]. PEPCK-C has been more widely studied for its key role in gluconeogenesis [15]. PEPCK-M was demonstrated to potentiate but not replace PEPCK-C-mediated gluconeogenesis [16]. PEPCK-M was also shown to play a critical role in the survival program initiated upon stress during metabolism in cancer cells [17]. Under low glucose conditions, *PCK2* expression is up-regulated in lung cancer cells [18, 19]. Glutamine is used for the production of PEP under glucose-limited conditions via the activity of PEPCK-M [19]. Glutamine-derived PEP is used as a biosynthetic intermediate for tumor cell proliferation [19]. Therefore, glucose deprivation may stimulate re-wiring of the TCA cycle to promote glucose-independent cell proliferation via the activation of PEPCK-M [19]. PEPCK-M is overexpressed in lung cancer cells compared to normal lung tissues and is activated for cell growth under glucose-depleted conditions [18, 19]. However, its role in other cancers is still largely unknown.

Several studies have shown that mTOR and its downstream signals are activated in pNETs [5-7]. Tuberous sclerosis complex 2 (TSC2) and phosphatase and tensin homolog (PTEN), two key inhibitors of the Akt/mTOR pathway, were found to be down-regulated in a majority of pNETs, and their low expression was correlated with shorter disease-free survival and overall survival [5]. mTOR inhibitors such as rapamycin or everolimus (RAD001) have been shown to successfully inhibit the proliferation of pNET cell lines [5]. In addition, everolimus (RAD001) has been used to treat patients with advanced pNETs, and it demonstrated anti-tumor effects with prolonged progression-free survival in phase II and III trials [10, 20, 21]. Rapamycin, an mTOR inhibitor, up-regulates or down-regulates the expression of some genes similar to the effect of glutamine, leucine and glucose deprivation. The genes involved in the TCA cycle can be up-regulated by rapamycin treatment, whereas the expression of genes that synthesize or use amino acids can be down-regulated by rapamycin treatment. The anti-proliferative effect of rapamycin has been shown to be due to the down-regulation of the activity of S6 Kinase 1 (S6K1) and eukaryotic translation-initiation factor 4E (eIF4E)-binding protein 1 (4E-BP1), and is likely a result of its capacity to mimic a starvation signal [22]. PEPCK-M links TCA cycle intermediates and glycolytic pools, whereas the intermediates may be derived from amino acids, mostly from glutamine. It is interesting to investigate whether the expression of PEPCK-M modulates the effect of mTOR inhibitors on pNET cells. In this study, we evaluated the expression of PEPCK-M in pNET patients and delineated the role of PEPCK-M in the growth of and response to mTOR inhibitors in pNET cells.

## RESULTS

### PEPCK-M is differentially expressed in pancreatic NETs (pNETs)

**Figure 1:**
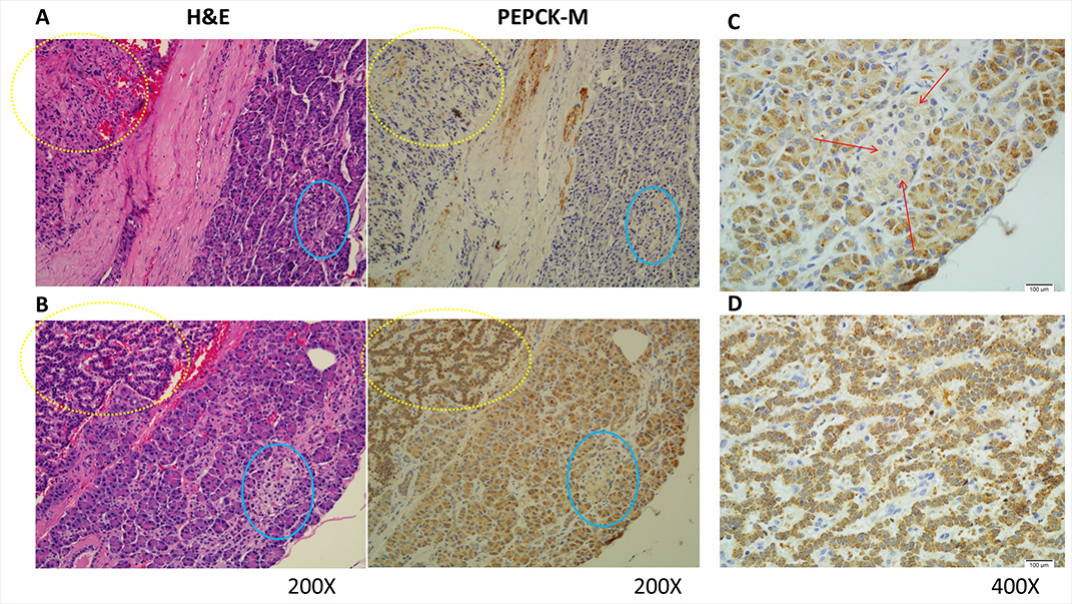
Representative hematoxylin and eosin (H&E) staining and immunohistochemical staining patterns for PEPCK-M in pNET tumor cells are shown (200X) **(A)** H&E and PEPCK-M staining for patient No. 2. PEPCK-M expression was weak (1+) in both tumor and non-neoplastic islet cells. **(B)** H&E and PEPCK-M staining for patient No. 20. PEPCK-M expression was strong (3+) in the tumor cells but weak (1+) in the non-neoplastic islet cells. The tumor cells are marked within yellow dashed circles, and the islet cells are marked within blue circles. **(C)** Magnified images (400X) of the islet cells marked by red arrows in Figure 1B. **(D)** Magnified images (400X) of the tumor cells in Figure 1B.

We evaluated PEPCK-M expression in pNET patients by immunohistochemistry. PEPCK-M was universally expressed in the cytosol. High expression (2+ and 3+) of PEPCK-M was observed in 12 (57%) of 21 pNET tumors. The other 9 (43%) pNET tumors had low expression of PEPCK-M (1+ and 0). In addition, we found that PEPCK-M was generally highly expressed in the normal pancreatic acini but weakly expressed in the normal islet and ductal cells in these patients. The PEPCK-M expression patterns in normal and tumor cells of two representative cases are shown in Figure 1A and 1B. Figure 1C and 1D show the magnified images of PEPCK-M staining in the islet cells and tumor cells, respectively, in Figure 1B. The expression patterns of PEPCK-M in the normal and tumor tissues of the 21 patients are listed in Supplementary Table 1. We did not find any association between age, sex, tumor grade, stage and survival of pNET patients with PEPCK-M expression, probably due to the limited number of cases. However, we found that PEPCK-C was highly expressed in 20 of 21 pNET tumors, with high expression in normal islet cells but low expression in normal acinar and ductal cells (Supplementary Table 2). The expression patterns of PEPCK-C in the two patients whose tumors are shown in Figure 1A and 1B are shown in Supplementary Figure 1A and 1B, respectively. The role of the variable PEPCK-M expression in pNETs merits further investigation.

### Knockdown of PCK2 inhibits the proliferation of pNET cells and enhances the sensitivity of pNET cells to mTOR inhibitors

**Figure 2:**
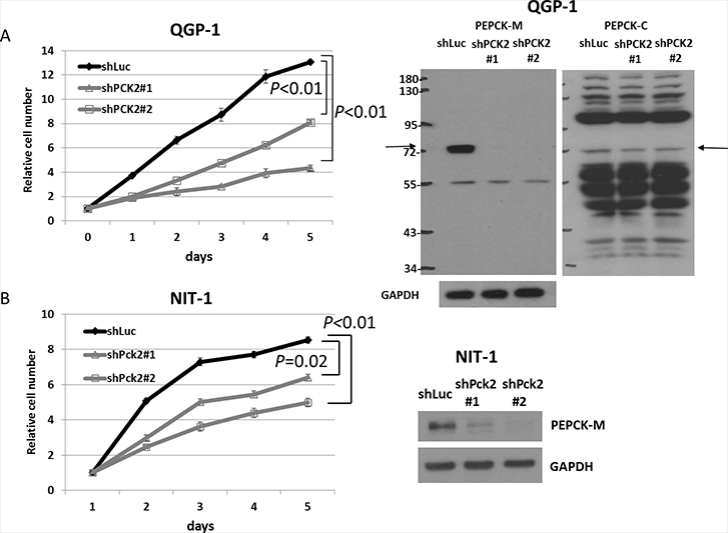
Cell proliferation curves of QGP-1 and NIT-1 cells with shRNA against *PCK2* and *Pck2* and vector control *Luc* from day 1 to day 5 **(A)** (Left) QGP-1 cells with knockdown of *PCK2* (2 clones, shPCK2#1 and shPCK2#2) had significantly lower rates of proliferation than QGP-1 cells infected with vector control (shLuc). (Right) The protein expression of PEPCK-M and PEPCK-C in QGP-1 cells with and without *PCK2* knocked down. **(B)** NIT-1 cells with knockdown of *Pck2* (2 clones, shPck2#1 and shPck2#2) had significantly lower rates of proliferation than NIT-1 cells infected with vector control (shLuc).

**Figure 3:**
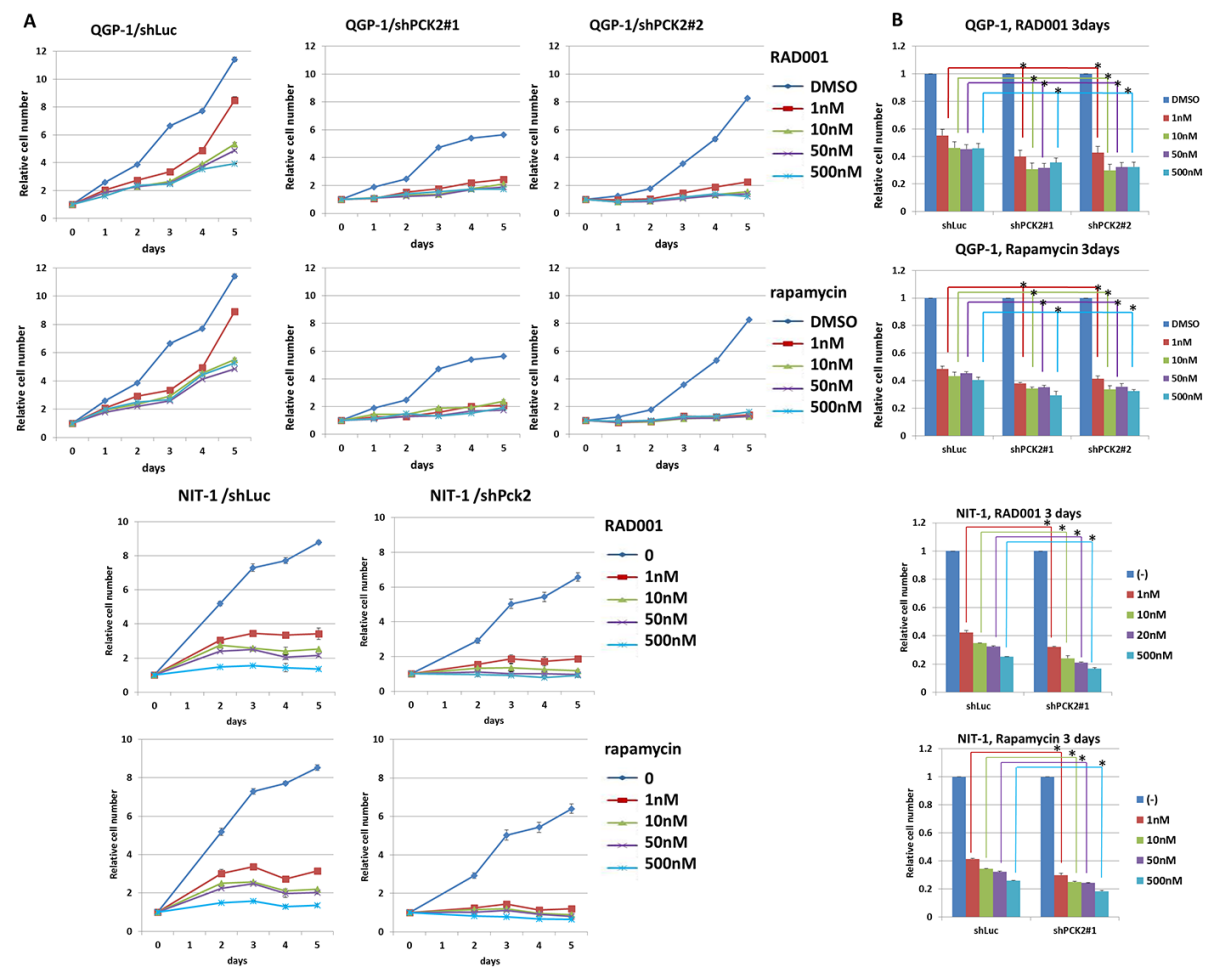
Cell proliferative curves and survival rate of QGP-1 and NIT-1 cells with or without knockdown of *PCK2* or *Pck2* and treatment of mTOR inhibitors, rapamycin or RAD001 **(A)** The cell proliferative rate was reduced by RAD001 and rapamycin for both QGP-1 and NIT-1 cells infected with vector control (shLuc). The cell proliferative rate of QGP-1 and NIT-1 cells was further decreased by RAD001 or rapamycin when *PCK2* was knocked down (shPCK2 and shPck2). **(B)** The survival rate of QGP-1 and NIT-1 cells treated with RAD001 or rapamycin for 3 days is shown. The survival of QGP-1/shPCK2 was significantly poorer than that of QGP-1/shLuc in low and higher dose of RAD001 or rapamycin. The similar result was also seen in NIT-1 cells. T test, ^*^ *P*<0.05.

We evaluated the effect of PEPCK-M on the proliferation of pNET cells. Knockdown of *PCK2* was achieved using a lentiviral approach in two pNET cell lines, human QGP-1 and mouse NIT-1, and stable clones were established. Growth of QGP-1 cells was significantly reduced in those with *PCK2* knocked down using 2 different *PCK2* shRNAs (QGP-1/shPCK2#1, QGP-1/shPCK2#2) compared with QGP-1 cells infected with shLuc (QGP-1/shLuc), as shown in Figure 2. PEPCK-M protein was significantly suppressed by the two shRNAs, whereas PEPCK-C (Figure 2) and pyruvate carboxylase (Supplementary Figure 2) were not significantly changed in QGP-1 cells by knockdown of *PCK2*. PEPCK-M was found to be present in the mitochondria but not cytosol (Supplementary Figure 2). In addition, cell proliferation was significantly retarded by knockdown of *Pck2* using 2 different *Pck2*-specific shRNAs in NIT-1 cells. Western blots and cell proliferation curves are shown in Figure 2. We assessed the effect of PEPCK-M on the anti-proliferative response of mTOR inhibitors in pNET cell lines. QGP-1 and NIT-1 cells infected with lentivirus expressing shPCK2 (or shPck2) and shLuc were treated with rapamycin or everolimus at indicated concentrations (0, 1, 10, 50 and 500 nM) for 5 days. Cell proliferation was assessed by methylene blue and WST1 assays. Cell proliferation curves of the two cell lines are shown in Figure 3A. When compared to vehicle control (DMSO), rapamycin and everolimus inhibited the proliferation of QGP-1 and NIT-1 cells with *PCK2* knocked down more significantly than that of cells infected with shLuc control, as shown in Figure 3B. We evaluated the protein expression of mTOR and its downstream targets, 4EBP1 and S6k, for QGP-1/shLuc and QGP-1/shPCK2 cells treated with or without RAD001 or rapamycin. Phosphorylation of 4EBP1 was reduced to a greater extent by RAD001 or rapamycin in QGP-1/shPCK2 cells than in QGP-1/shLuc cells (Supplementary Figure 3). These results may explain the effect of PCK2 on the sensitivity of QGP-1 cells to mTOR inhibitors.

### PEPCK-M affects genes associated with mTORC1 and glycolysis pathways

**Figure 4:**
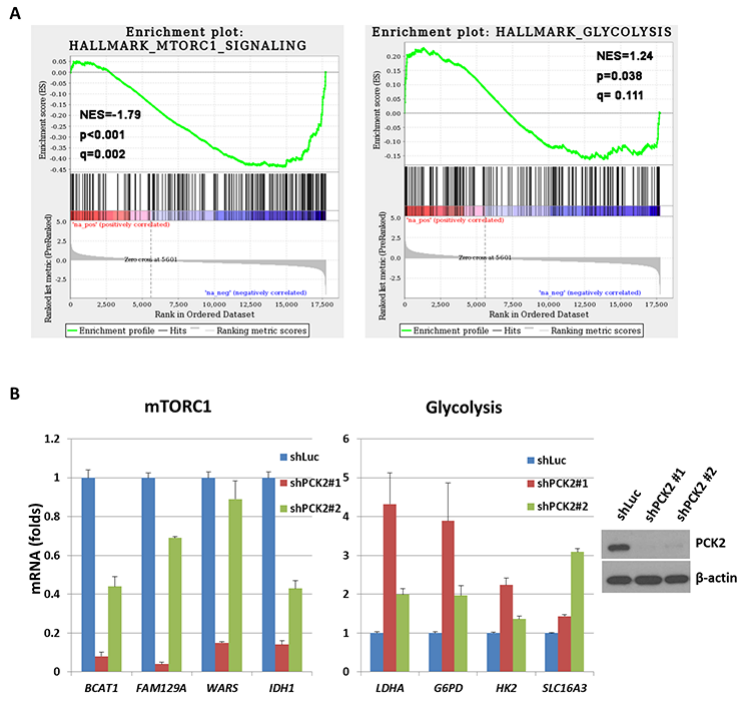
The result of gene expression microarray for QGP-1 cells with knockdown of *PCK2* compared with QGP-1 infected with vector control (shLuc) **(A)** Two representative significantly enriched gene sets in the differential expression profiles. Hallmark_mTORC1_sugnaling is negatively enriched (down-regulated) and Hallmark_Glycolysis is positively enriched (up-regulated) in general in QGP-1 cells with *PCK2* knocked down. **(B)** the ratio of quantitative RT-PCR for 4 down-regulated genes, *BCAT1, FAM129A, WARS, and IDH1* in mTORC1-related gene sets and 4 up-regulated genes, *LDHA, G6PD, HK2, and SLC16A3,* in Glycolysis-related gene sets in QGP-1 cells with knockdown of *PCK2* (shPCK2) relative to that in QGP-1 cells without knockdown of *PCK2* (shLuc).

To probe the mechanisms underlying the reduced cell proliferation and increased sensitivity to mTOR inhibitors upon silencing of *PCK2* in pNET cells, we performed gene expression microarray analyses to obtain differential gene expression profiles between cells transfected with shPCK2 or vector control (shLuc). The up-regulated and down-regulated gene sets in QGP-1/shPCK2 cells and QGP-1/shLuc cells are listed in Supplementary Table 3. Using GSEA, we found two gene sets, including mTORC1-related and glycolysis-related genes (Figure 4A), that were among the significantly enriched gene sets in the differential expression profiles, with the former being negatively enriched (down-regulated) and the latter positively enriched (up-regulated). To validate the reliability of the array experiments and following pathway analysis, quantitative RT-PCR was performed on some genes selected by the leading edge analysis of GSEA, including *BCAT1*, *FAM129A, WARS, IDH1, SLC16A3*, *HK2*, *LDHA* and *G6PD*. The results confirmed the down-regulation of *BCAT1*, *FAM129A, WARS,* and *IDH1* in the mTORC1-related gene sets and up-regulation of *SLC16A3*, *HK2*, *LDHA* and *G6PD* in the glycolysis-related gene set upon knockdown of *PCK2*, as shown in Figure 4B. The protein level of BCAT1 and IDH1 was decreased in both QGP-1 clones with *PCK2* knocked down, whereas the protein level of LDHA and MCT4 (SLC16A3) was increased in one QGP-1 clone with PCK2 knocked down (Supplementary Figure 4). The protein expression of mTOR and mTOR downstream targets S6K and 4EBP1 was not significantly reduced. These results suggest that mTORC1 signaling and glycolysis pathways were indeed negatively and positively affected, respectively, by shRNA against *PCK2*.

### PEPCK-M regulates glycolysis and mitochondrial oxidative phosphorylation in pNET cells

**Figure 5:**
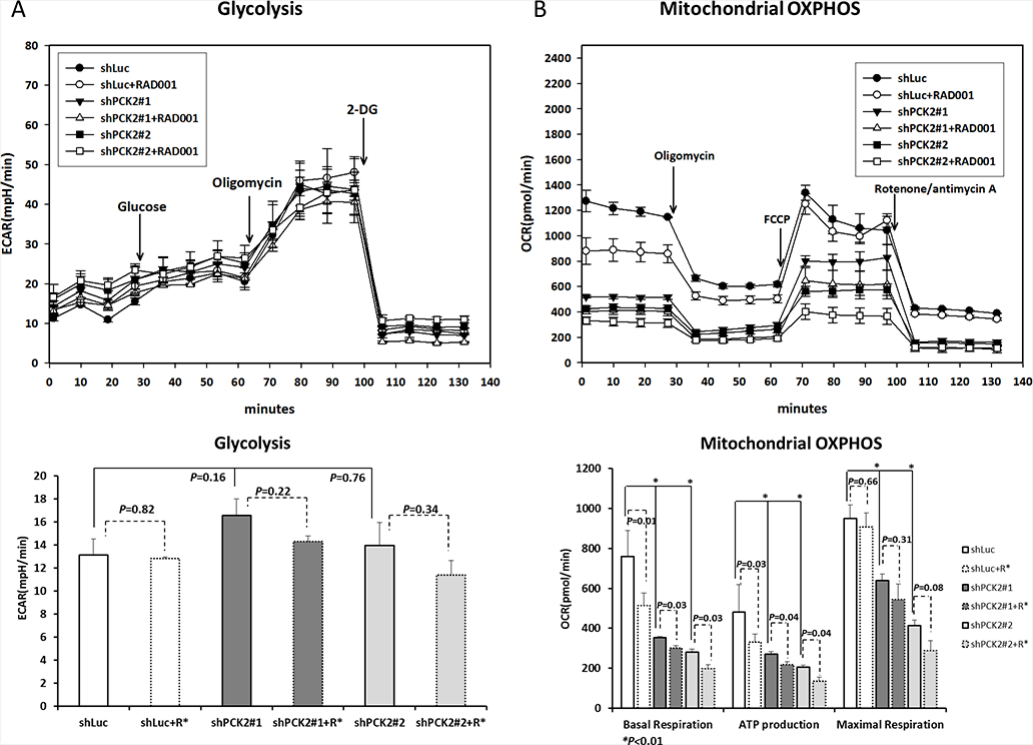
Glycolysis and mitochondrial oxidative phosphorylation (OXPHOS) analysis of QGP-1 cells with and without knockdown of *PCK2* and with or without treatment with RAD001 **(A)** The change of extracellular acidification rate (ECAR) in of QGP-1/shLuc cells was not significantly changed by treatment with 50 nM of RAD001. The ECAR was slightly higher in QGP-1/shPCK2 (#1 and #2) cells than that in QGP-1/shLuc cells. The ECAR in QGP-1/shPCK2 cells was slightly reduced by RAD001 treatment. The lower figure shows the bar graph of ECAR in QGP-1/shLuc and GQP-1/shPCK2 cells with and without RAD001 treatment. **(B)** The oxygen consumption rate (OCR), including basal respiration, ATP production, and maximal respiration, in mitochondria were reduced in QGP-1/shPCK2 cells than that in QGP-1/shLuc cells. RAD001 induced a reduction in OCR, including basal respiration, ATP production but not maximal respiration, to QGP-1/shLuc and QGP-1/shPCK2 cells. The lower figure shows the bar graph of OCR in QGP-1/shLuc and QGP-1/shPCK2 cells with and without RAD001 treatment.

Because the up-regulation of genes involved in the glycolysis pathway was noted in QGP-1 cells with *PCK2* knocked down, we measured glycolysis (extracellular acidification rate (ECAR)) and function of the mitochondria (oxygen consumption rate (OCR)) for QGP-1 cells with and without *PCK2* knocked down. The ECAR was slightly increased in QGP-1/shPCK2 cells, albeit not statistically significant, whereas the OCR including basal respiration, ATP production, and maximal respiration were significantly lower in the two QGP-1/shPCK2 clones (shPCK2#1 and shPCK2#2) than that in QGP-1/shLuc cells, as shown in Figure 5A and 5B. We also measured the lactate level in QGP-1 cells with or without *PCK2* knocked down. The lactate level was significantly increased in QGP-1 cells with PCK2 knocked down (Supplementary Figure 5). These results suggest that the knockdown of *PCK2* promotes glycolysis but reduces mitochondrial OXPHOS in QGP-1 cells. Because PEPCK-M links TCA cycle intermediates and glycolytic pools via the conversion of mitochondrial oxaloacetate into PEP, we evaluated the proliferation of QGP-1 cells cultured in medium with normal, low or no glucose. The proliferation rate of QGP-1 cells with PEPCK-M (QGP-1/shLuc cells) was more reduced under conditions of low (1 mM and 0.1 mM of glucose) or no glucose than cells under conditions of normal glucose (10 mM of glucose), as shown in Supplementary Figure 6. These results suggest that glycolysis is important for the proliferation of QGP-1 cells. The proliferation rates of the two QGP-1/shPCK2 clones were low in both medium with normal glucose and that with low/no glucose, as shown in Supplementary Figure 6. The protein expression of BCAT1 in the mTORC1-related gene set and LDHA and MCT4 in the glycolysis-related gene set in QGP-1/shLuc and QGP-1/shPCK2 cells was reduced when the cells were cultured in medium without glucose (Supplementary Figure 7). Although knockdown of *PCK2* led to increased glycolysis, the cell proliferation rate was as high as that observed in cells grown in medium with normal glucose level. When the cells were cultured in medium with high (10 mM), normal (2 mM) or no (0 mM) glutamine, the proliferation rate of QGP-1/shPCK2 cells was not significantly increased in medium with high level of glutamine. However, the cells proliferated at a low rate when cultured in medium without glutamine irrespective of *PCK2*, as shown in Supplementary Figure 8. Knockdown of *PCK2* in QGP-1 cells led to a down-regulation of *BCAT1, IDH1* and *WARS*, which are associated with biosynthesis, the TCA cycle and tRNA processing, and reduced mitochondrial OXPHOS. Taken together, these results suggest that mitochondrial OXPHOS and glycolysis are both important for the proliferation of QGP-1 cells.

### PEPCK-M regulates glycolysis and mitochondrial oxidative phosphorylation in pNET cells in response to an mTOR inhibitor

Because the anti-proliferative effect of mTOR inhibitors against QGP-1 cells was enhanced after *PCK2* knockdown compared to shLuc control, we measured the ECAR and OCR of cells with and without treatment of RAD001 at 50 nM for 4 hours. Figure 5A and 5B show that the ECARs of QGP-1/shLuc cells were not significantly different between the cells with and without RAD001 exposure. However, a slight reduction, although not statistically significant, of ECAR was noted in QGP-1/shPCK2 cells treated with RAD001. In addition, a significant reduction in lactate level in QGP-1/shPCK2 (#1 and #2) cell but not in QGP-1/shLuc cells was also noted as a result of RAD001 (Supplementary Figure 5). Taken together, the changes in ECAR and lactate in QGP-1 cells by *PCK2* knockdown demonstrate the effect of *PCK2* on glycolysis as well as on mTOR inhibitor-induced alteration in glycolysis in QGP-1 cells. By contrast, the OCRs, including basal respiration and ATP production but not maximal respiration, in the mitochondria of both QGP-1/shLuc and QGP-1/shPCK2 cells were reduced to a similar extent when the cells were treated with RAD001.

### PEPCK-M attenuates the survival of QGP-1 cells treated with the anti-glycolysis agent 2-DG

**Figure 6:**
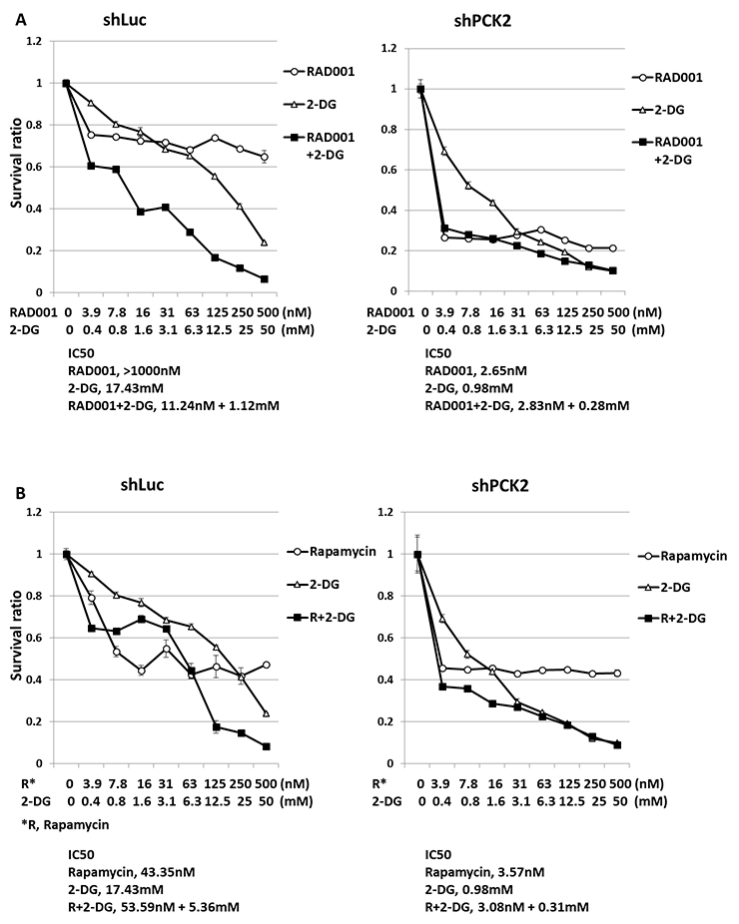
The survival rate of QGP-1 cells with and without knockdown of *PCK2* treated with two mTOR inhibitor, 2-DG alone and combination of the two agents **(A)** The survival rate of QGP-1/shLuc cells treated with RAD001, 2-DG alone and combination of RAD001 with 2-DG in various concentrations. Combination of both drugs induced further reduced survival of QGP-1/shLuc cells. The survival rate of QGP-1/shPCK2 cells can be reduced by RAD001 or 2-DG alone in each dose level when compared with the survival of QGP-1/shLuc cells treated with the indicated agent. **(B)** The survival rate of QGP-1/shLuc cells treated with rapamycin, 2-DG alone and combination of rapamycin with 2-DG in various concentrations. Rapamycin or 2-DG alone induced reduced survival rate of QGP-1/shLuc cells. Combination of both drugs at higher dose induced further reduction in survival rate of QGP-1/shLuc cells. The survival rate of QGP-1/shPCK2 cells can be reduced by rapamycin alone when compared with the survival of QGP-1/shLuc cells treated with rapamycin. The survival rate of QGP-1/shPCK2 cells can be further reduced by combination of rapamycin and 2-DG than treatment with each agent alone at lower doses. The IC50 of each drug was listed below the figure.

Because increased glycolysis was noted in QGP-1 cells with knockdown of *PCK2*, we evaluated the impact of PEPCK-M on the survival rate of QGP-1 cells treated with mTOR inhibitors or 2-DG alone or in combination for 3 days. Figure 6A shows that QGP-1/shPCK2 cells had worse survival than QGP-1/shLuc cells when treated with RAD001 or 2-DG alone. The IC50 of RAD001 in QGP-1/shLuc cells was >1000 nM, and it was reduced to 2.65 nM in QGP-1/shPCK2 cells. The IC50 of 2-DG in QGP-1/shLuc cells was 17.43 mM, and it was reduced to 0.98 mM in QGP-1/shPCK2 cells. The survival rate of both cells was further reduced by the combination of RAD001 and 2-DG, particularly for the QGP-1/shLuc cells. The enhanced sensitivity of QGP-1 cells to RAD001 and 2-DG alone or in combination by the knockdown of *PCK2* was also observed by the use of the other construct of shPCK2 (shPCK2#2), as shown in Supplementary Figure 9A. These results were also obtained in NIT-1 cells (Supplementary Figure 9B). A similar result was noted for QGP-1/shLuc and QGP-1/shPCK2 cells treated with rapamycin and 2-DG alone or in combination, as shown in Figure 6B. The synergistic or additive effect of the combination of rapamycin and 2-DG on the inhibition of proliferation of QGP-1/shLuc cells was more significant at higher doses of the drugs.

## DISCUSSION

PEPCK-M is encoded by *PCK2*. According to the analysis of the results obtained from The Cancer Genome Atlas (TCGA) project, elevated expression of *PCK2* occurs in various human tumors, including thyroid, urinary tract, breast and lung cancers [19]. However, the role of aberrant expression of PEPCK-M in cancers is not well understood. Mendez-Lucas et al. have shown that knockdown of *PCK2* in MCF7 and HeLa cells induces a reduction in glucose consumption, lactate production and glycolytic flux and results in growth retardation of these cells. *PCK2* gene transcription can be up-regulated by amino acid limitation, ER stress inducers or ER stress. PEPCK-M protects MCF7 cells from apoptosis induced by glutamine deprivation and by the ER stress inducer thapsigargin [16]. Using quantitative PCR, Leithner et al. have shown that *PCK2* is significantly over-expressed in tumors compared to normal lung tissues of non-small cell lung cancer (NSCLC) patients. Tissues from 14 of 20 (70%) NSCLC patients were positively stained with PEPCK-M at variable proportions by immunohistochemistry. In their study, *PCK2* expression was enhanced under low-glucose conditions. In addition, lung cancer cells have been shown to convert lactate to pyruvate via PEPCK-M under low-glucose conditions. Knockdown of *PCK2* or addition of the PEPCK inhibitor 3-Mercaptopicolinate (3-MP) to lung cancer cells significantly enhanced glucose depletion-induced apoptosis of these cells [18]. Park et al. have shown that the down-regulation of PEPCK-M leads to reduced proliferation of the colon cancer cell line SNU-C4 and increased survival of SNU-C4 cells treated with a combination of 5-FU and radiation. Rectal cancer patients with low protein expression of PEPCK-M showed poor responses to preoperative 5-FU-based radiation therapy [23]. In our study, we found that PEPCK-M was highly expressed in the tumors from 12 of 21 (57%) pNET patients, whereas all the islet cells of these patients had weak expression of PEPCK-M. Although Stark et al. reported that PEPCK-M is present in INS-1 832/13, a pancreatic islet cell line, rat islets and mouse islets and that PEPCK-C is absent in all the three cell lines [24], the extent of PEPCK-M expression in those cells was not mentioned. In our study, PEPCK-C was highly expressed in 20 of 21 pNET tumors and all identified normal islet cells in 15 pNET patients (Supplementary Table 2). Furthermore, PEPCK-M was up-regulated, but PEPCK-C was not changed under glucose deprivation, as shown in Supplementary Figure 10. Therefore, we speculate that PEPCK-C has a weak effect on the pathogenesis or tumor progression of pNETs. However, Seenappa et al. reported that PEPCK-C protein expression was markedly reduced in the cancer cell line HepG2 cells but reduced at a lower extent in fibroblasts under culture conditions lacking glucose. However, PEPCK-M protein expression was slightly elevated in HepG2 cells and not altered in fibroblasts under the same conditions [25]. In summary, the regulation of PEPCK-C and PEPCK-M in response to glucose deprivation is different among different cancer types and not the same as that of normal cells. *In vitro*, we verified the impact of PEPCK-M on the proliferation of pNET cell lines similar to the effect observed in breast, cervical, lung and colon cancer cell lines [17, 18, 23]. The mechanism of PEPCK-M in promoting cell proliferation is not well understood. Mendez-Lucas et al. have demonstrated that knockdown of PEPCK-M in MCF7 cells induced reduction in glucose consumption, lactate production, and glycolytic flux and resulted in reduced proliferation without apoptosis [17]. By contrast, we found that glycolysis was up-regulated and mitochondrial OXPHOS was down-regulated in QGP-1 cells by knockdown of *PCK2.* In our study, we delineated the metabolic regulation of pNET cells by PEPCK-M and showed that QGP-1 cells proliferate using glycolysis and mitochondrial OXPHOS pathways, which was demonstrated by the lower proliferative rate of QGP-1 cells in medium containing low or no glucose and by knockdown of *PCK2*. The QGP-1/shLuc cells had the highest proliferation rate in medium with a normal glucose level, whereas the cells could still proliferate in medium without glucose, although at a lower rate. These results suggest that QGP-1 cells proliferate using glycolysis, either through anerobic or aerobic pathways, and mitochondrial OXPHOS with intermediates derived from amino acids, such as glutamine. When *PCK2* was knocked down, the proliferation rate of QGP-1 cells was further reduced in glucose-free medium than that in normal glucose-containing medium. The proliferation of QGP-1 cells with *PCK2* knocked down was reduced in culture medium with high or normal levels of glutamine (Supplementary Figure 8), which suggests that PCK2 is involved in the TCA cycle because glutamine is a source of TCA cycle metabolites. The attenuated proliferation can still be observed by supplementation of glutamine in QGP-1 cells with *PCK2* knocked down, which suggests that *PCK2* regulates the TCA cycle. Our data showed that the knockdown of *PCK2* reduced the OCR, i.e., mitochondrial OXPHOS, but increased the ECAR, i.e., glycolysis and glycolytic capacity, of QGP-1 cells. This result suggested that PEPCK-M deficiency partially rescues the proliferative capacity of QGP-1 cells via up-regulation of glycolysis when glucose is available in the environment. The high level of OCR observed in QGP-1/shPCK2 cells following antimycin and rotenone treatment (Figure 5B) indicates high levels of non-mitochondrial respiration. These results are probably unique for pNET cells, as pNET is a hypervascular tumor and distinct from adenocarcinoma cells, which have been studied thoroughly. Therefore, PEPCK-M is a potential therapeutic target for pNETs. The PEPCK inhibitor 3-MP has been shown to inhibit proliferation of lung cancer cells by targeting PEPCK-M, and a similar effect was observed in pNET cells in our study (Supplementary Figure 11).

Montal et al. have demonstrated the association between PEPCK and mTORC1 in colon cancer cells. Because relative gene expression of *PCK2* was only present in less than 3% of colon cancers according to TCGA database, their study focused on PEPCK-C. According to their study, PEPCK was shown to promote proliferation and activation of mTORC1 in Colo205 cells, a human colon adenocarcinoma cell line. Knockdown of PEPCK in Co1o205 cells caused a significant reduction in TCA cycle intermediates derived from glutamine, glycolysis and flux of glucose into the TCA cycle, which resulted in reduced proliferation rate of Colo205 cells. Knockdown of PEPCK also attenuated the sensitivity of Colo205 cells to rapamycin [26]. The biological mechanism for the association between PEPCK-M and mTOR is not well understood. mTOR has been shown to regulate glycolysis and mTOR inhibitors have been shown to suppress glycolysis of various cancers [27-31]. However, Jesus et al. have shown an opposite effect of mTOR inhibition, whereby rapamycin increased glucose consumption and decreased mitochondrial complex III expression in human Sertoli cells [32]. In our study, PEPCK-M was shown to attenuate the sensitivity of pNET cells to mTOR inhibitors. PEPCK-M affects the mTOR inhibitor-induced change in ECAR and OCR of QGP-1 cells. Increased mTORC1 activity was noted by Montal et al., [26] when PEPCK-C was silenced in Colo205 cells; this result raises the question of whether PEPCK-C affects the activity of mTORC1 in QGP-1 cells with PEPCK-M knocked down. However, we have shown that PEPCK-C protein expression was not changed after knockdown of *PCK2* in QGP-1 cells. Therefore, it can be considered that the reduction in the mTORC1 pathway in QGP-1 cells with *PCK2* knocked down is not associated with PEPCK-C. These results suggest that the prosurvival effect of PEPCK-M deficiency in QGP-1 cells mediated by up-regulating glycolysis is negated by the inhibition of mTOR, which leads to further reduction in proliferation of QGP-1 cells treated with mTOR inhibitors. The prominent reduction of phosphorylated 4EBP1 in QGP-1/shPCK2 cells compared to that in QGP-1/shLuc cells likely explains the reduction of glycolysis by RAD001 in QGP-1/shPCK2 cells but not in QGP-1/shLuc cells. In addition, we showed that genes associated with the mTORC1 pathway were down-regulated by knockdown of *PCK2* in QGP-1 cells. Among them, *BCAT1* was significantly down-regulated by knockdown of *PCK2* in QGP-1 cells. *BCAT1* encodes branched-chain amino acid transaminase 1, which transaminates branched chain amino acids (BCAAs) to branched-chain ɑ-ketoacids (BCKAs). After transamination, BCKAs are further catabolized to intermediates of the TCA cycle including acetyl coenzyme A and succinyl-coenzyme A for subsequent oxidization, which is essential for cellular metabolism and growth [33-35]. Down-regulation of *BCAT1*, which is associated with catabolism mediated by the TCA cycle, in QGP-1 cells with knockdown of *PCK2* probably explains the reduced mitochondrial OXPHOS and reduced cell proliferation. Taken together, PEPCK-M regulates the mTORC1 pathway in pNET cells probably by regulating glycolysis and mitochondrial OXPHOS, which, to the best of our knowledge, is a novel finding.

Because approximately half of the pNET patients have high expression of PEPCK-M and we identified the impact of PEPCK-M on pNET, targeting PEPCK-M alone or in combination with an mTOR inhibitor may be a potential therapeutic option for pNETs. There is still no PEPCK-M inhibitor available for clinical use. However, based on the finding that both glycolysis and mitochondrial OXPHOS are important for the proliferation of pNET cells, inhibiting glycolysis may be a possible therapeutic approach for pNET. The synergistic effect of mTOR inhibitors and 2-DG in reducing the proliferation of QGP-1 cells was demonstrated in our study, particularly when in the presence of PEPCK-M (QGP-1/shLuc cells). For QGP-1 cells with PEPCK-M knocked down (QGP-1/shPCK2 cells), this synergistic effect is less significant, and mTOR inhibitor or 2-DG alone can inhibit the proliferation of QGP-1/shPCK2 cells more effectively than that of QGP-1/shLuc cells. Although we have found that QGP-1 cells were sensitized to the glycolysis inhibitor 2-DG following knockdown of PCK2 (Figure 6), the cells were less sensitive to glucose deprivation (Supplementary Figure 6) with regard to cell proliferation. 2-DG was used as a glycolysis inhibitor; however, as well as inhibiting glycolysis it also caused other metabolic changes, for example, it was found to decrease TCA metabolites and increase metabolites of the pentose phosphate pathway in leukemia cells [36]. 2-DG was also demonstrated to induce cell cycle arrest and apoptosis in colorectal cancer cells independent of glycolysis inhibition [37]. Therefore, the anti-proliferative effect of 2-DG on QGP-1/shLuc and QGP-1/shPCK2 cells was probably through the inhibition of glycolysis and other mechanisms. Because 2-DG has been tested for clinical use [38], the combination of 2-DG with an mTOR inhibitor for pNET treatment may be a potential option.

In conclusion, PEPCK-M promotes the proliferation of pNET cells probably by regulating glycolysis and mitochondrial OXPHOS. Knockdown of *PCK2* induces the down-regulation of genes in the mTORC1 pathway and up-regulation of genes in the glycolysis pathway. PEPCK-M attenuates the anti-proliferative response of pNET cells to mTOR inhibitors. The combination of the glycolysis inhibitor 2-DG with mTOR inhibitors synergistically or additively inhibits the proliferation of pNET cells. Therefore, targeting PEPCK-M or the glycolytic pathway may be a potential therapeutic approach for the treatment of pNETs and warrants further investigation.

## MATERIALS AND METHODS

### Cell lines and reagents

QGP-1, a human pNET cell line, was purchased from Japanese Collection of Research Bioresources (JCRB, Tokyo, Japan). NIT-1, a mouse pNET cell line, was purchased from Bioresource Collection and Research Center (BCRC, Hsinchu, Taiwan). The passage number of the two cell lines used in this study was less than 15. The QGP-1 cell line that we used was sent to the Center for Genomic Medicine of National Cheng Kung University for genotyping on Jun 2016, and the result showed the same STR DNA profile as those in the JCRB database. QGP-1 and NIT-1 cells were cultured in RPMI-1640 medium (Hyclone, South Logan, Utah, USA) and F12 Kaighn’s medium (Gibco, Grand Island, NY, USA), respectively, containing 10% fetal calf serum and antibiotics. The shRNAs targeting *PCK2* and *Pck2* were obtained from the National RNAi Core Facility of Academic Sinica (Taipei, Taiwan). Rapamycin and RAD001 were purchased from LC laboratories (Woburn, MA, USA) and Selleckchem (Munich, Germany), respectively. 2-Deoxy-D-glucose (2-DG) was purchased from Seahorse Bioscience (Billerica, MA, US). The primary antibody against PEPCK-M was purchased from GeneTex (GTX114919, USA). Antibodies against PEPCK-C (sc-74825) and GAPDH were purchased from Santa Cruz (Texas, US). Antibody against β-actin was purchased from Millipore (MA, US).

### Stable cell establishment

Stable gene knockdown in QGP-1 and NIT-1 cells was achieved by a lentiviral infection system according to the instructions provided by the National RNAi Core Facility of Academic Sinica. We replaced the virus-containing medium with fresh medium 24 hours after lentiviral infection, and the cells were treated with puromycin to select the infected cells. Stable cell clones were confirmed by western blotting to check the expression of PEPCK-M.

### Cell proliferation and survival assay (WST1 assay or methylene blue colorimetric assay)

QGP-1 cells (1 × 10^4^ per well for 96-well plate and 4 × 10^4^ per well for 24-well plate) were treated with different dosages of rapamycin, RAD001, 2-DG, or combinations of the above agents. The treated cells were incubated for 72, 96 or 120 hours at 37°C in 5% CO_2_-containing incubator. Cell proliferation was determined by incubating the cells with WST-1 Cell Proliferation Reagent (Clontech, CA, US) for 1 hour. The absorbance was measured at 450 nm using a SpectraMax M5 microplate reader (Molecular Devices, US). The survival rate of cells was determined by the absorbance of cells with the indicated dosage divided by the absorbance of cells not treated with drugs or treated with DMSO. Cell proliferation was also determined by methylene blue colorimetric assay. Briefly, 0.5% methylene blue solution was incubated with adherent cells in microplates for 1 hour and then removed by washing with PBS. Finally, 1% sarcosine was added to each well of the microplates to dissolve the methylene blue, and the growth curves were determined according to absorbance at 595 nm. The cell proliferation rate of the different clones infected with shLuc or shPCK2 was set to 1 on day 0, and the proliferation rate of the cells from day 1 to day 5 was calculated by dividing the absorbance at 595 nm on each day to the absorbance at 595 nm on day 0. The survival rate of the cells treated with DMSO was set at 1 (100%), and the survival rate of the cells treated with the indicated drugs was calculated by dividing the absorbance at 595 nm of cells exposed to drugs to the absorbance at 595 nm of cells exposed to DMSO. Each data point was measured in triplicate, and the result was presented as the mean ± standard error.

### Western blotting

Lentivirus-infected cells were harvested in lysis buffer. Equal amounts of protein were subjected to SDS-PAGE. Proteins were transferred to PVDF membranes, and the blots were incubated with different primary antibodies against targets including PEPCK-M, PEPCK-C, BCAT1, IDH1, LDHA, MCT4, pyruvate carboxylase, COX4, S6K, p-S6K, 4EBP1, p-4EBP1, mTOR, p-mTOR, GAPDH and β-actin. Enhanced chemiluminescence reagents were used to detect the protein bands on the membrane and visualized by UVP biospectrum 600.

### Expression array and gene set enrichment analysis (GSEA)

Gene expression microarray analyses were performed according to the protocol described in our previous study [39] to obtain the differential expression profile of stable shPCK2 clone versus shLuc (vector and scramble control) clone. GSEA [40] was applied to analyze gene sets or molecular pathways enriched in the differential expression profiles using log2 ratio as the ranking metric, where the ratio was defined as the normalized intensity of a specific gene in shPCK2 cells divided by that in control cells. Java GSEA desktop application downloaded from the GSEA website (http://software.broadinstitute.org/gsea/downloads.jsp) was used for GSEA implementation.

### Quantitative RT-PCR

RNA was extracted with TriPure Isolation Reagent (Roche, Mannheim, Germany) according to the manufacturer’s instructions. RNA quantification was performed using a NanoDrop 2000 (Thermo Scientific, Wilmington, USA) spectrophotometer. Two micrograms of total RNA was reverse transcribed using High-Capacity cDNA Reverse Transcription Kit (Applied Biosystems, Foster City, CA). Quantitative RT-PCR was performed with Fast SYBR Green Master Mix (Applied Biosystems, Foster City, CA) with ABI 7500 Fast system (Applied Biosystems, Foster City, CA). The primers used for the detection of *BCAT1, LDHA, G6PD, SLC16A3, HK2, IDH1, FAM129A, WARS, and ACTB* are listed in Supplementary Table 4. The real-time cycler conditions were as follows: initial activation at 95°C for 10 min, 45 cycles of melting at 95°C for 15 sec, and annealing/extension at 60°C for 1 min. The Ct value used for the real-time PCR quantification was defined as the PCR cycle number that crossed an arbitrarily chosen signal threshold in the log phase of the amplification curve. To verify the fold change of the target gene expression, calculated Ct values were normalized to Ct values of β-actin (*ACTB*) amplified from the same sample (ΔCt = Ct_target_ - Ct_β-actin_), and the 2^-ΔΔCt^ method was used to calculate fold change. Each sample was measured in triplicate, and the mean fold change and standard error (S.E.) between shLuc and shPCK2 are presented.

### Detection of glycolysis and mitochondrial oxidative phosphorylation

QGP-1 cells were seeded in XF 24-well cell culture microplates (Seahorse Bioscience, Billerica, MA, USA) at 2.0 X 10^4^ cells/well (0.32 cm^2^) in 100 μl growth medium and then incubated at 37°C with 5% CO_2_ for 20-24 hours. The culture medium was replaced with assay medium (unbuffered RPMI supplemented with 2 mM glutamine and 10% serum, pH 7.4) and cultured for 1 hour before the assay. The metabolic response, extracellular acidification rate (ECAR) and oxygen consumption rate (OCR) were measured using a Seahorse XF24 Extracellular Flux Analyzer (Seahorse Bioscience), which measures glycolysis and OXPHOS in real time according to the manufacturer’s protocol. The data were reported in pmol/min for OCR and mpH/min for ECAR. Each experiment was carried out in triplicate, and the results are presented as the mean ± S.E.

### Immunohistochemistry and scoring

Tumors and adjacent non-neoplastic tissues from 21 pNET patients who received surgical intervention, post-operative treatment, and follow-up at National Cheng Kung University Hospital from Aug 2006 to Dec 2015 were selected and analyzed in this study. Immunostaining was performed using Leica Bond-Max automatic immunostainer (Leica, Bannockburn, IL) following 25 min incubation at room temperature in Bond™ Epitope Retrieval Solution 1 (Leica Biosystems, Catalog No. AR9961). Slides were incubated with 1:100 dilution of anti-PEPCK-C antibody and 1:200 dilution of anti-PEPCK-M antibody for 1 hour at room temperature. Paraffin-embedded sections of normal renal tubule cells and human breast cancer cells were included as positive controls for PEPCK-C and PEPCK-M, respectively. The negative controls had the primary antibodies replaced with PBS. The expression of PEPCK-M and PEPCK-C was rated semi-quantitatively based on the staining intensity and staining percentage. The staining intensity was scored as “0”, “1+”, “2+”, and “3+” for “Negative”, “Weak”, “Moderate” and “Strong” staining, respectively. The staining percentage was scored as ranging from 0 to 100% for tumor cells.

### Statistical analysis

The difference in proliferation rates between the different clones was analyzed by t-test. The differences in survival rates of cells treated with various drugs compared to control were analyzed by t-test. The differences in ECAR, OCR and lactate levels between different QGP-1 clones and between cells treated with or without RAD001 were analyzed by t-test. The correlation between clinical data and immunohistochemical staining for PEPCK-M expression was determined using Fisher’s exact test.

## SUPPLEMENTARY MATERIALS FIGURES AND TABLES






