# Supplementary material for: Mitochondrial phosphoenolpyruvate carboxykinase (PEPCK-M) regulates the cell metabolism of pancreatic neuroendocrine tumors (pNET) and de-sensitizes pNET to mTOR inhibitors
Source: Oncotarget. 2017 Oct 9;8(61):103613–25. doi: 10.18632/oncotarget.21665 (PMC5732754; doi:10.18632/oncotarget.21665)
Supplement: Supplementary file 1 [file oncotarget-08-103613-s001.pdf]

Mitochondrial phosphoenolpyruvate carboxykinase (PEPCK-M) regulates the cell metabolism of pancreatic neuroendocrine tumors (pNET) and de-sensitizes pNET to mTOR inhibitors

SUPPLEMENTARY MATERIALS

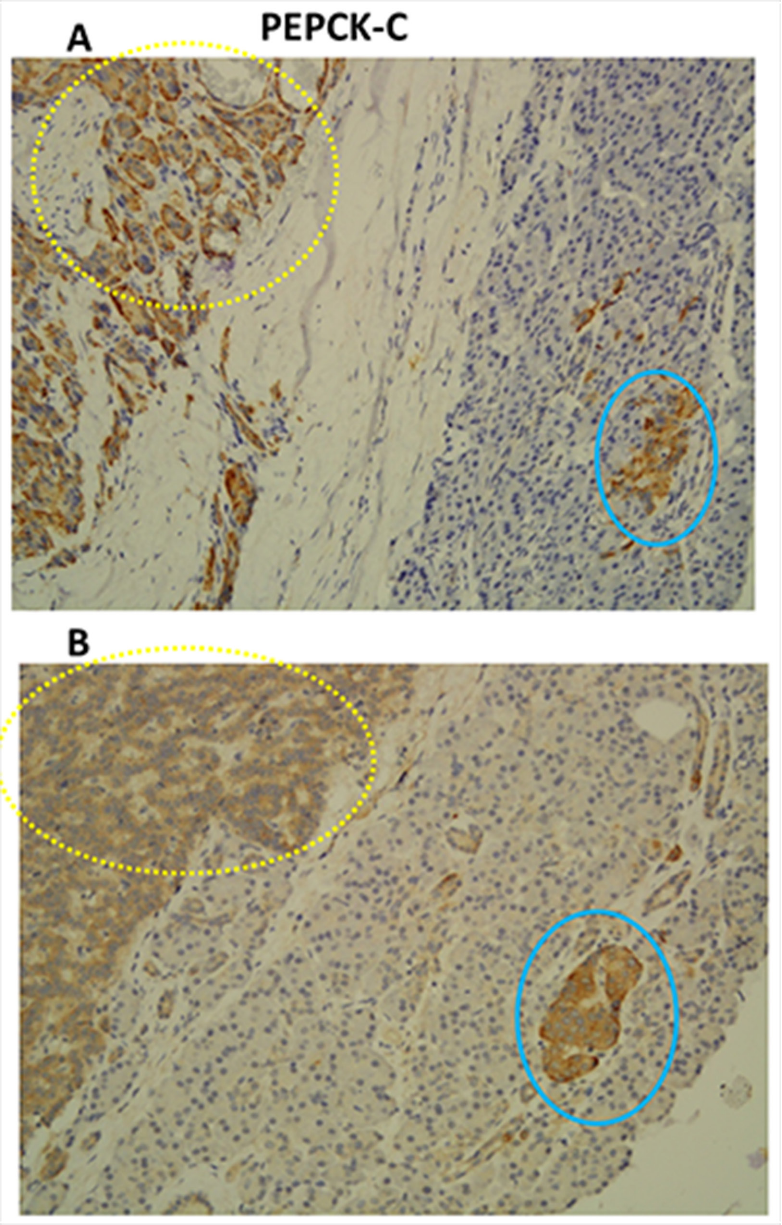

Supplementary Figure 1: The PEPCK-C expression in pNET patients. **(A)** The expression pattern of PEPCK-C in the tumor and islet cells of the patient shown in Figure 1A. **(B)** The expression pattern of PEPCK-C in the tumor and islet cells of the patient shown in Figure 1B. The tumor cells are marked within yellow dashed circles, and the islet cells are marked within blue circles.

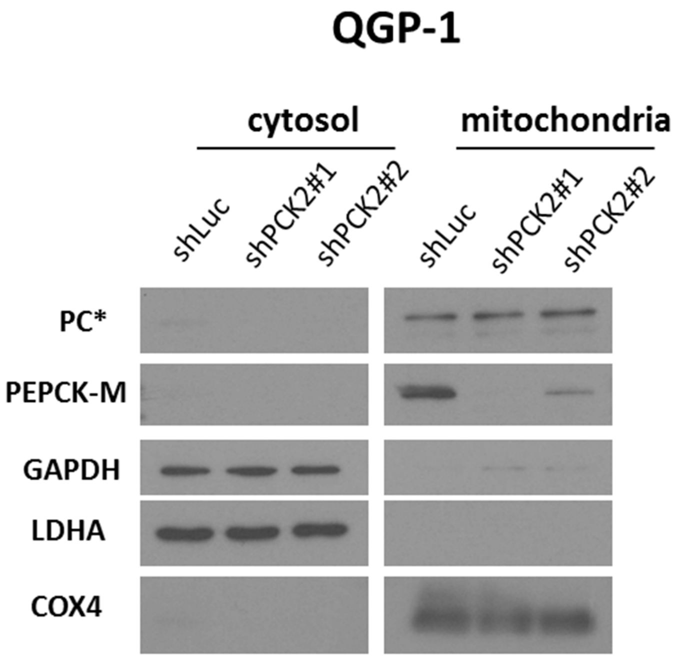

Supplementary Figure 2: The protein expression of pyruvate carboxylase in QGP-1/shLuc and QGP-1/shPCK2 (#1 and #2) cells were not significantly different. Pyruvate carboxylase and PEPCK-M were located in mitochondrial.

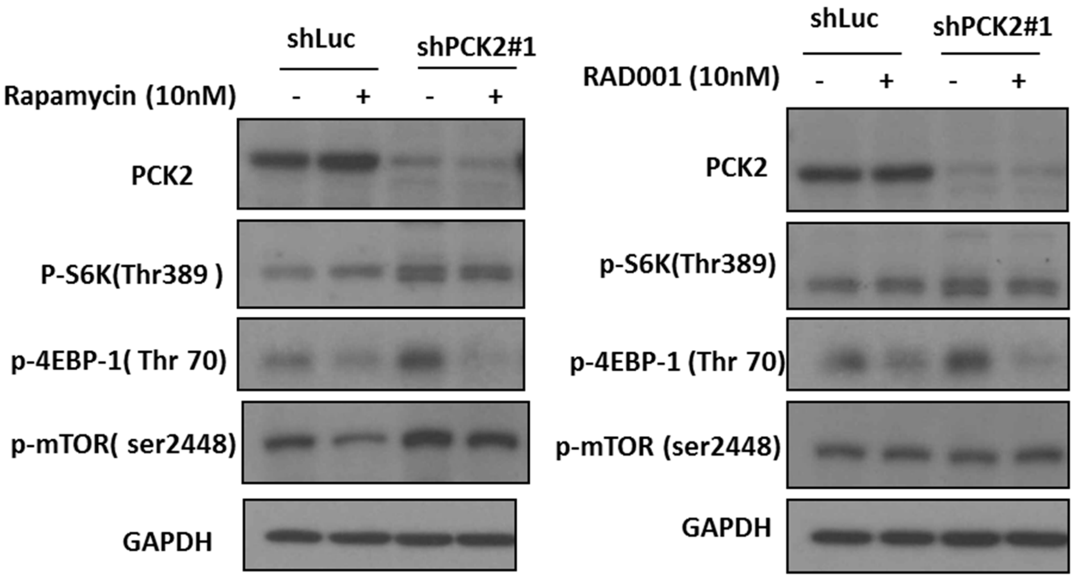

Supplementary Figure 3: The protein expression of phosphorylated mTOR and its downstream targets S6K and 4EBP-1 of QGP-1/shLuc and QGP-1/shPCK2#1 cells with or without Rapamycin or RAD001 treatment.

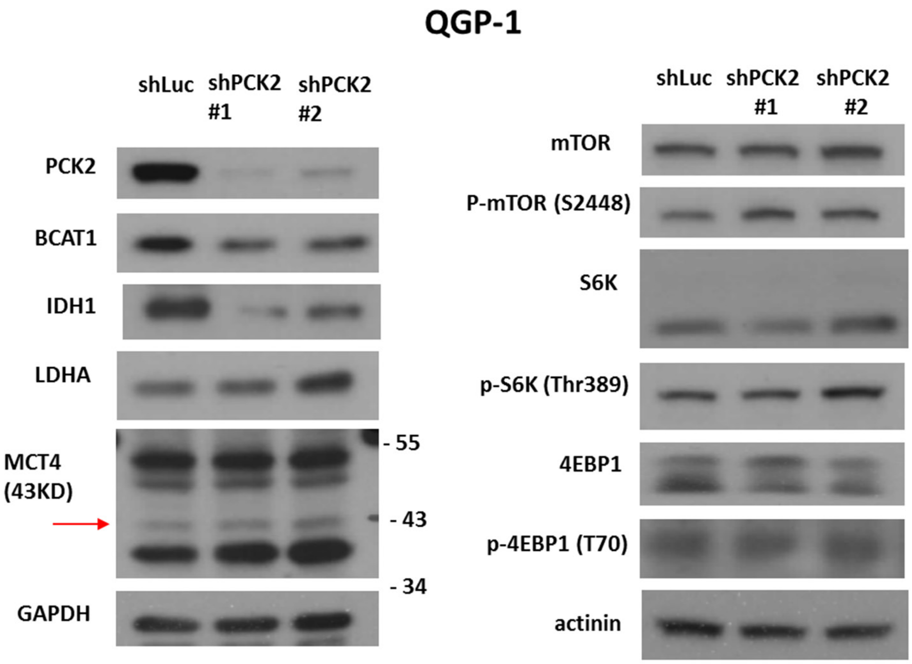

Supplementary Figure 4: The protein expression of mTORC1-related genes, BCAT1 and IDH1, glycolysis-related genes, LDHA and MCT4, and mTOR and the downstream targets of mTOR in QGP-1/shLuc and QGP-1/shPCK2 (#1 and #2) cells.

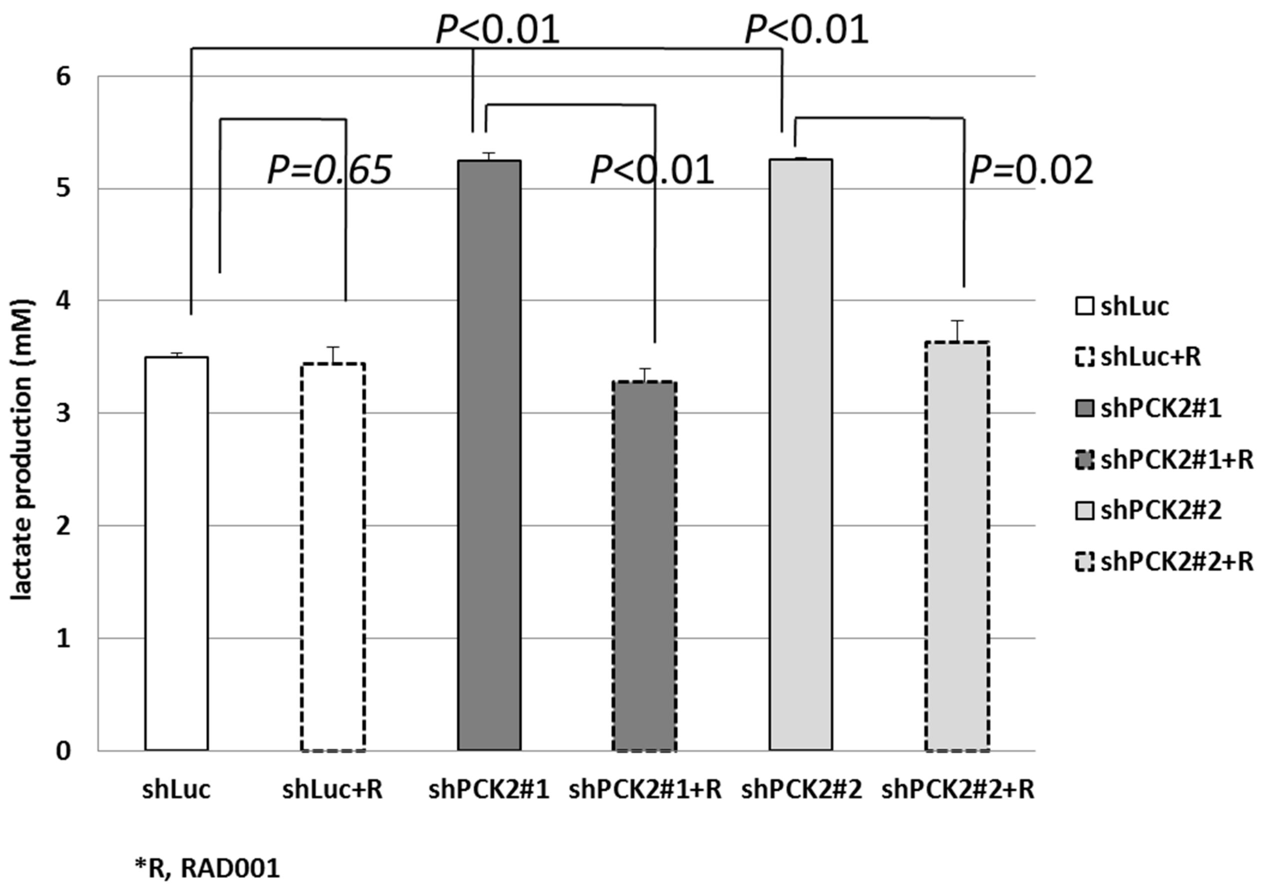

Supplementary Figure 5: The lactate level of QGP-1/shLuc and QGP-1/shPCK2 (#1 and #2) cells with or without RAD001 treatment.

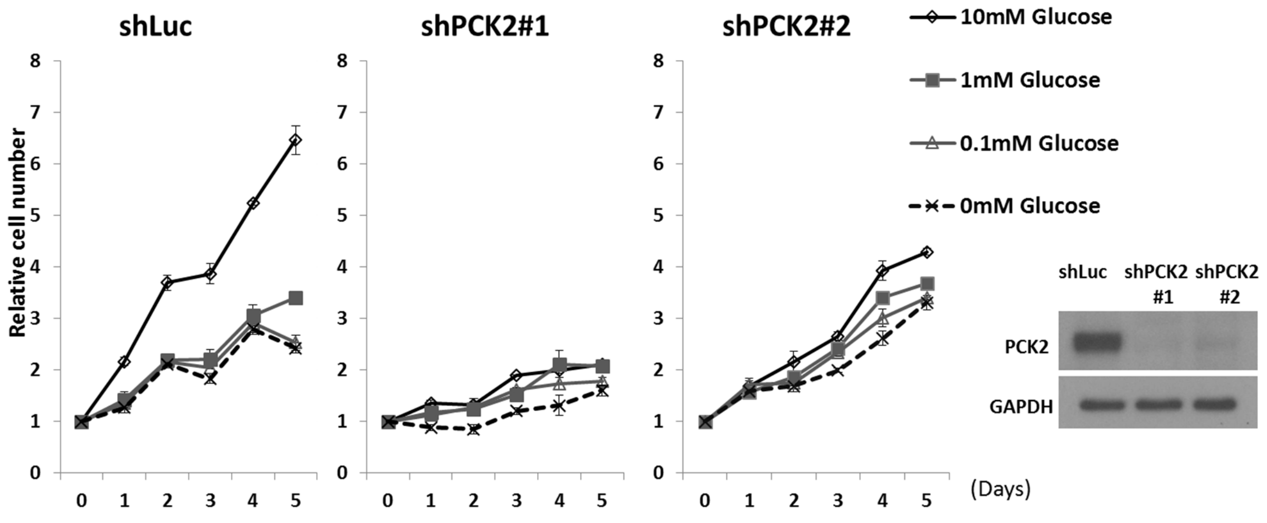

Supplementary Figure 6: The proliferative rate of QGP-1/shLuc and QGP-1/shPCK2 (#1 and #2) cells cultured under normal (10mM), low (1 and 0.1mM) or no (0mM) glucose–contained medium.

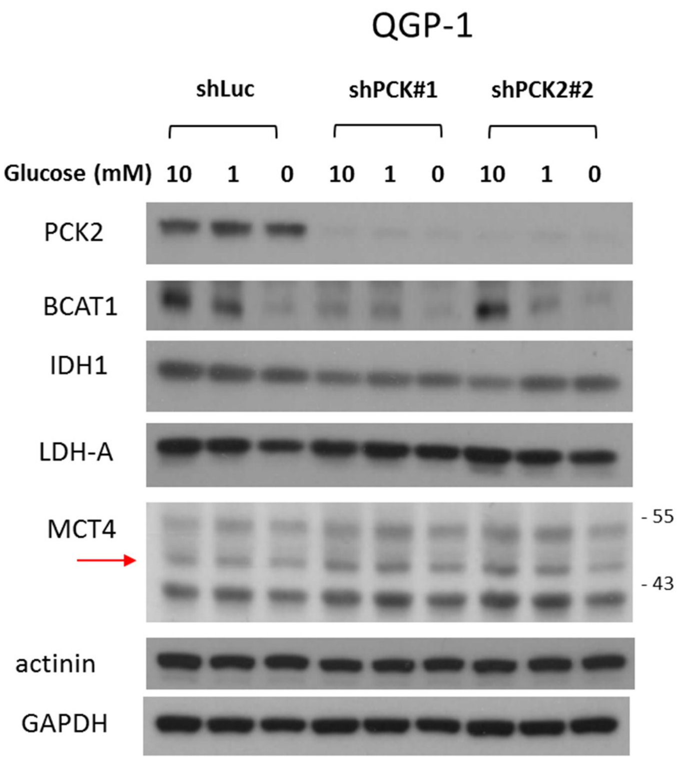

Supplementary Figure 7: The protein expression of mTORC1-related genes, BCAT1 and IDH1, and glycolysis-related genes, LDHA and MCT4, in QGP-1/shLuc and QGP-1/shPCK2 (#1 and #2) cells cultured under normal (10mM), low (1mM), and no glucose-contained medium.

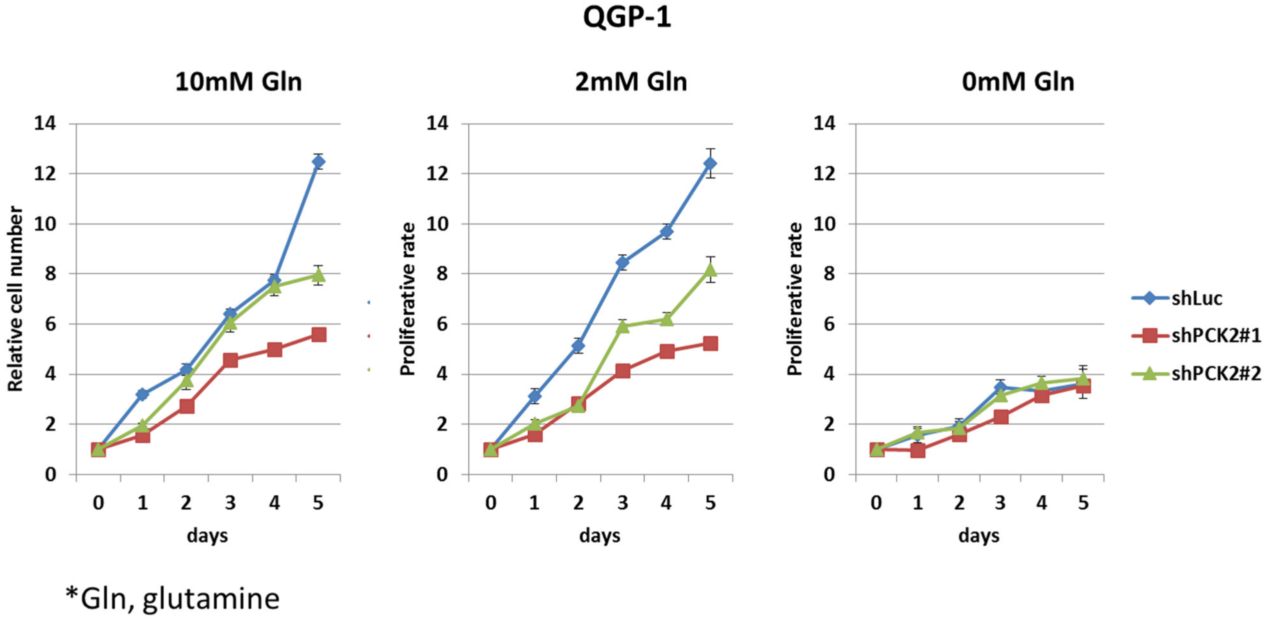

Supplementary Figure 8: The proliferative rate of QGP-1/shLuc and QGP-1/shPCK2 (#1 and #2) cells cultured under high (10mM), normal (2mM) or no (0mM) glutamine–contained medium.

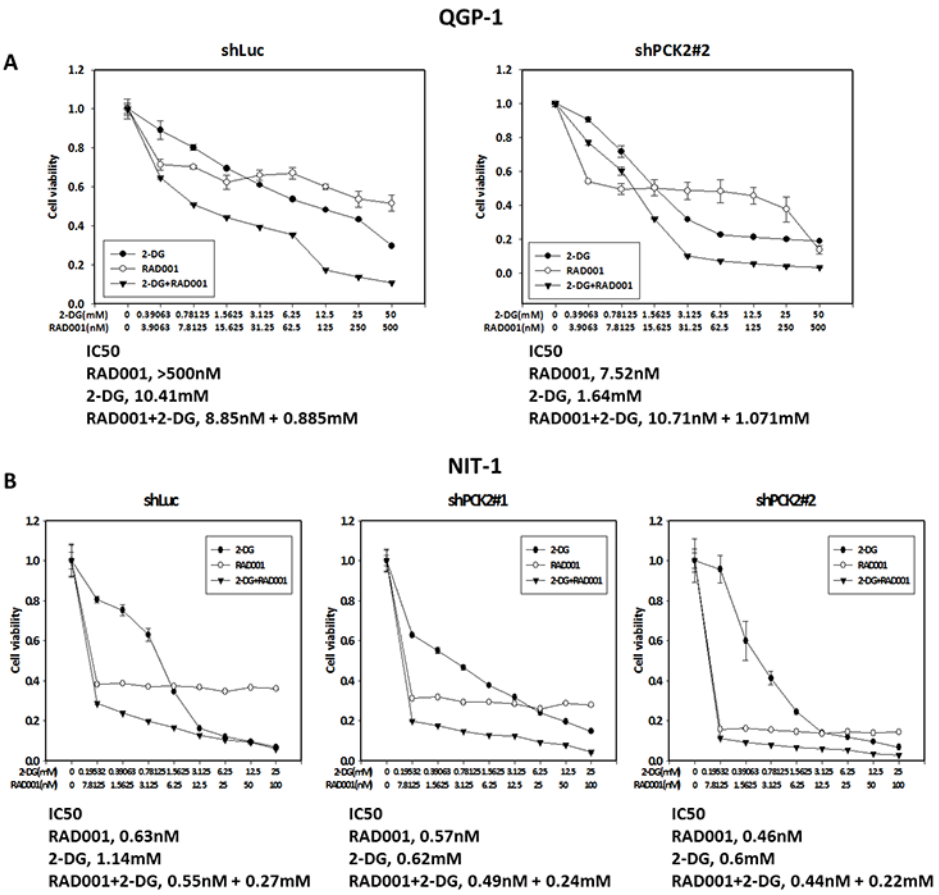

Supplementary Figure 9: The cell viability of QGP-1 and NIT-1 cells with or without knockdown of PCK2 treated with RAD001 alone, 2-DG alone or combination of RAD001 and 2-DG. **(A)** The cell viability of QGP-1/shLuc and QGP-1/shPCK2 #2 cells treated without or with indicated doses of 2-DG alone, RAD-001 alone or combination of 2-DG and RAD001. **(B)** The cell viability of NIT-1/shLuc and NIT-1/shPck2 (#1 and #2) cells treated without or with indicated doses of 2-DG alone, RAD-001 alone or combination of 2-DG and RAD001.

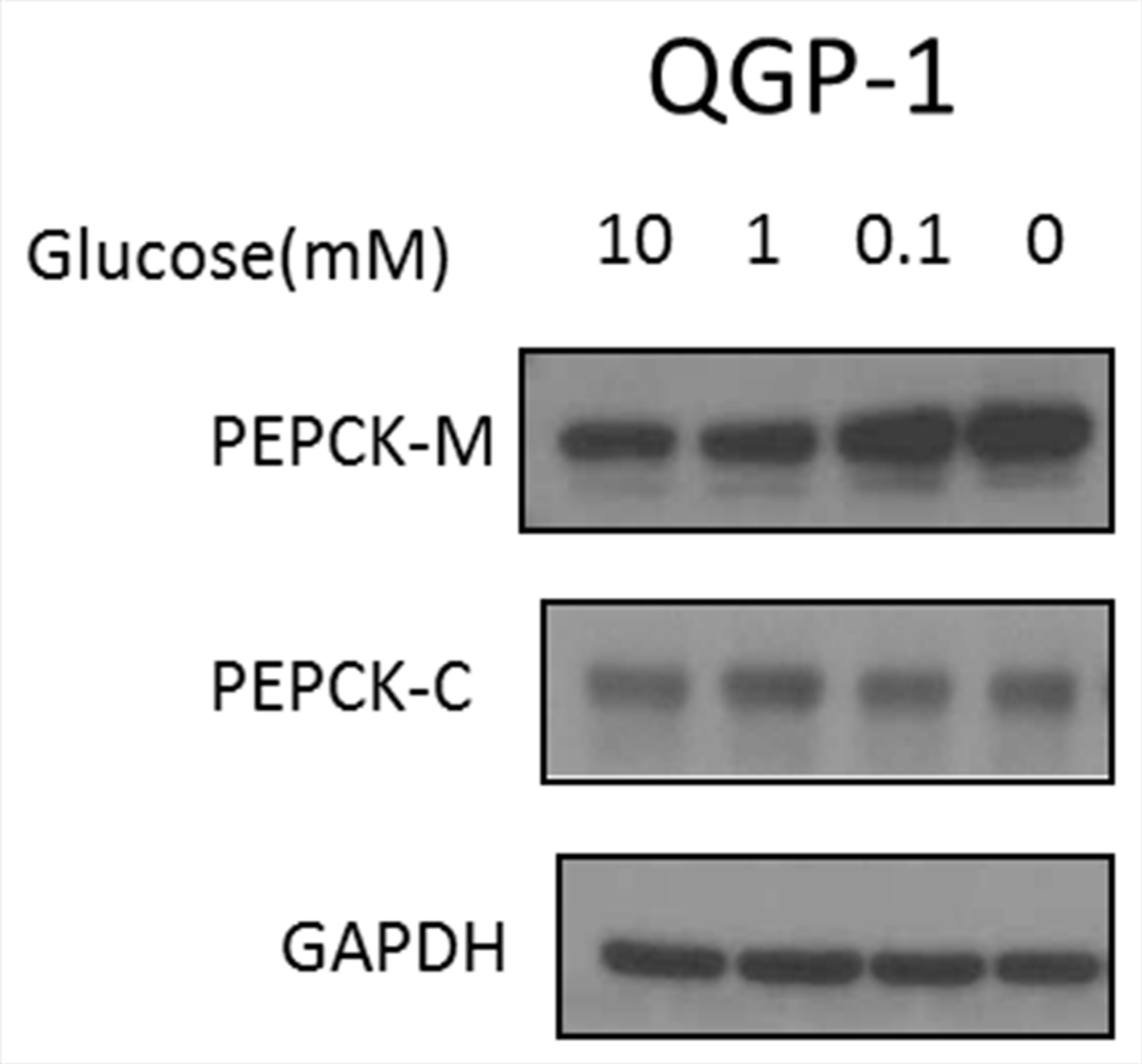

Supplementary Figure 10: The protein expression of PEPCK-M and PEPCK-C in QGP-1 cells under normal (10mM), low (1, and 0.1mM) or no (0) glucose culture condition. The PEPCK-M expression was increased under low or no glucose condition but the PEPCK-C was not affected by glucose concentration.

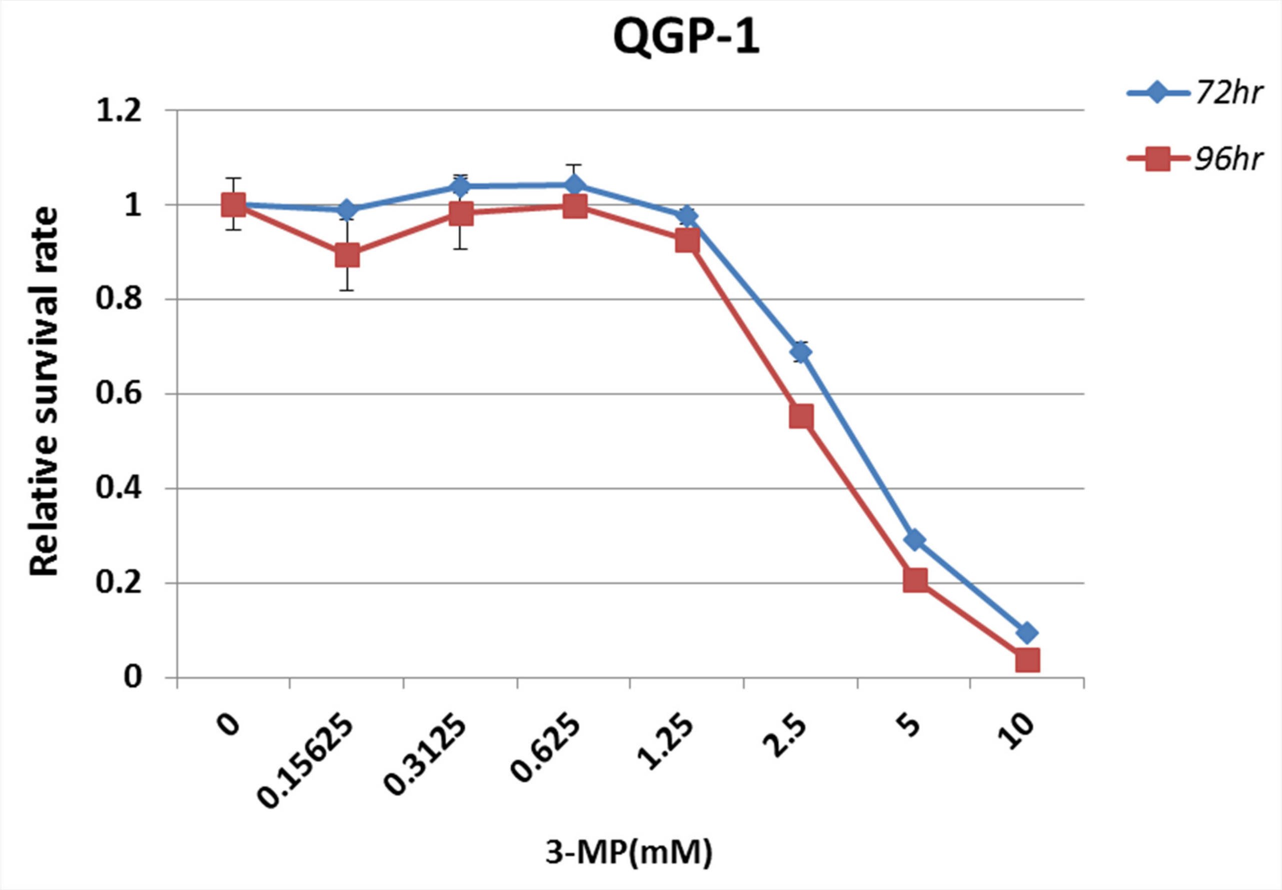

Supplementary Figure 11: The survival rate of QGP-1 cells treated with or without indicated doses of PEPCK inhibitor, 3-MP, for 72 and 96 hours.

Supplementary Table 1: Expression scoring and percentage of PEPCK-M in 21 pNET patients

See Supplementary File 1

Supplementary Table 2: Expression scoring and percentage of PEPCK-C in 21 pNET patients

patient No.	Tumor grade	normal acinar cells	normal islet cells	pNET	normal ductal cells
1	2	-	3+, 100%	1+, 100%	-
2	1	0	3+, 100%	3+, 80%	0
3	2	-	-	2+, 80%	-
4	1	0	2+, 100%	2+, 90%	0
5	2	1+, 80%	2+, 100%	2+, 100%	1+, 80%
6	1	-	-	2+, 100%	-
7	1	1+, 20%	3+, 100%	2+, 60%	1+, 20%
8	1	1+, 20%	3+, 80%	3+, 80%	1+, 20%
9	2	0	3+, 60%	3+, 60%	0
10	2	-	-	3+, 100%	1+, 60%
11	2	1+, 40%	3+, 100%	3+, 80%	1+, 80%
12	1	-	-	3+, 80%	-
13	1	0	2+, 100%	2+, 100%	0
14	1	1+, 10%	3+, 100%	3+, 100%	1+, 10%
15	1	-	-	3+, 100%	-
16	1	1+, 20%	2+, 90%	2+, 80%	1+, 40%
17	1	0	3+, 100%	2+, 80%	0
18	2	1+, 20%	3+, 100%	3+, 80%	1+, 20%
19	1	1+, 40%	3+, 100%	3+, 90%	1+, 40%
20	1	1+, 20%	3+, 100%	3+, 80%	1+, 20%
21	2	-	-	3+, 60%	-

-, cell not found
0, no expression

Supplementary Table 3: The up-regulated and down-regulated gene sets in QGP- 1/shPCK2 cells and QGP-1/shLuc cells

See Supplementary File 2

Supplementary Table 4: Sequences of primers for the indicated genes

Genes	Forward	Reverse
BCAT1	5’-CCAAAGCCCTGCTCTTTGTA-3’	5’-TGGAGGAGTTGCCAGTTCTT-3’
LDHA	5′-GGCCTCTGCCATCAGTATCT-3′	5′-GCCGTGATAATGACCAGCTT-3′
G6PD	5’-ACGTGATGCAGAACCACCTACTG-3’	5’-ACGACGGCTGCAAAAGTGGCG-3’
MCT4	5’-GTTGGGTTTGGCACTCAACT-3’	5’-GAAGACAGGGCTACCTGCTG-3’
HK2	5’-AACCATGACCAAGTGCAGAA-3’	5’-AGCCCTTTCTCCATCTCCTT-3’
IDH1	5’-GGTGACATACCTGGTACATAACTTTG-3’	5’-GTGTGCAAAATCTTCAATTGACTT-3’
FAM129A	5’-CCAGGAGTCAGAGGAAGAGAAG-3’	5’-GTTGCCACAGGATTCACCAC-3’
WARS	5’-CCTCATTCCATTTATTTTCACAAA-3’	5’-ATGACCAAGGGCACGTTAAA-3’
ACTB	5’-GCTGTGCTACGTCGCCCT-3’	5’-AAGGTAGTTTCGTGGATGCC-3’
