# Supplementary material for: Mitochondrial phosphoenolpyruvate carboxykinase (PEPCK-M) regulates the cell metabolism of pancreatic neuroendocrine tumors (pNET) and de-sensitizes pNET to mTOR inhibitors
Source: Oncotarget. 2017 Oct 9;8(61):103613–25. doi: 10.18632/oncotarget.21665 (PMC5732754; doi:10.18632/oncotarget.21665)
Supplement: Supplementary file 2 [file oncotarget-08-103613-s002.docx]

Supplementary Table 1.

Expression scoring and percentage of PEPCK-M in 21 pNET patients

| patient No. | Tumor  grade | normal acinar cells | normal islet cells | pNET | normal ductal cells |
| --- | --- | --- | --- | --- | --- |
| 1 | 2 | - | - | 1+, 80% | - |
| 2 | 1 | 3+, 20% | 1+, 60% | 1+, 20% | 1+, 60% |
| 3 | 2 | - | - | 3+, 20% | - |
| 4 | 1 | 2+, 40% | 1+, 60% | 3+, 40% | 1+, 60% |
| 5 | 2 | 2+, 80% | 1+, 100% | 3+, 80% | 1+, 100% |
| 6 | 1 | - | - | 1+, 60% | - |
| 7 | 1 | 2+, 60% | 1+, 40% | 1+, 60% | 1+, 40% |
| 8 | 1 | 3+, 20% | 1+, 40% | 1+, 60% | 1+, 40% |
| 9 | 2 | 2+, 20% | 1+, 40% | 1+, 40% | 1+, 40% |
| 10 | 2 | - | - | 3+, 80% | 1+, 100% |
| 11 | 2 | 3+, 60% | 1+, 100% | 1+, 100% | 1+, 100% |
| 12 | 1 | - | - | 2+, 60% | - |
| 13 | 1 | 2+, 60% | 1+, 80% | 2+, 40% | 1+, 80% |
| 14 | 1 | 2+, 80% | 1+, 100% | 3+, 80% | 1+, 100% |
| 15 | 1 | - | - | 2+, 90% | - |
| 16 | 1 | 3+, 90% | 1+, 100% | 2+, 100% | 1+, 100% |
| 17 | 1 | 2+, 60% | 1+, 40% | 3+, 60% | 1+, 60% |
| 18 | 2 | 3+, 80% | 2+, 60% | 2+, 90% | 1+, 80% |
| 19 | 1 | 3+, 80% | 1+, 100% | 1+, 80% | 1+, 100% |
| 20 | 1 | 3+, 60% | 1+, 100% | 3+, 90% | 1+, 100% |
| 21 | 2 | - | - | 1+, 20% | - |

-, cell not found

The correlation of PEPCK-M expression with age, sex, tumor grade, stage, and survival

|  | PEPCK-M | | |
| --- | --- | --- | --- |
|  | Low expression  N = 9  n (%) | High expression  N = 12  n (%) | *P* |
| Age, median | 54.0 | 50.0 | 0.60 |
|  |  |  |  |
| Sex |  |  |  |
| F | 3 (33.3) | 4 (33.3) | 1.00 |
| M | 6 (66.7) | 8 (66.7) |  |
|  |  |  |  |
| Grade |  |  |  |
| I | 5 (55.6) | 8 (66.7) | 0.67 |
| II | 4 (44.4) | 4 (33.3) |  |
|  |  |  |  |
| Stage |  |  |  |
| I + II | 5 (55.6) | 8 (66.7) | 0.67 |
| IV | 4 (44.4) | 4 (33.3) |  |
|  |  |  |  |
| Survival |  |  |  |
| 0 | 1 (11.1) | 1 (8.3) | 1.00 |
| 1 | 8 (88.9) | 11 (91.7) |  |

The *P* value was generated by Fisher’e Exact Test.
