# Supplementary material for: Mitochondrial phosphoenolpyruvate carboxykinase (PEPCK-M) regulates the cell metabolism of pancreatic neuroendocrine tumors (pNET) and de-sensitizes pNET to mTOR inhibitors
Source: Oncotarget. 2017 Oct 9;8(61):103613–25. doi: 10.18632/oncotarget.21665 (PMC5732754; doi:10.18632/oncotarget.21665)
Supplement: Supplementary file 3 [file oncotarget-08-103613-s003.docx]

Supplementary Table 3

Enriched gene sets in QGP-1/shPCK2 (up-regulated) vs QGP-1/shLuc

| Gene set | NES | p | q |
| --- | --- | --- | --- |
| [HALLMARK_COAGULATION](http://www.broadinstitute.org/gsea/msigdb/cards/HALLMARK_COAGULATION) | 2.15 | <0.001 | <0.001 |
| [HALLMARK_KRAS_SIGNALING_UP](http://www.broadinstitute.org/gsea/msigdb/cards/HALLMARK_KRAS_SIGNALING_UP) | 1.68 | <0.001 | 0.044 |
| [HALLMARK_ESTROGEN_RESPONSE_LATE](http://www.broadinstitute.org/gsea/msigdb/cards/HALLMARK_ESTROGEN_RESPONSE_LATE) | 1.67 | <0.001 | 0.03 |
| HALLMARK_EPITHELIAL_MESENCHYMAL_TRANSITION | 1.6 | <0.001 | 0.035 |
| [HALLMARK_KRAS_SIGNALING_DN](http://www.broadinstitute.org/gsea/msigdb/cards/HALLMARK_KRAS_SIGNALING_DN) | 1.57 | <0.001 | 0.03 |
| [HALLMARK_IL6_JAK_STAT3_SIGNALING](http://www.broadinstitute.org/gsea/msigdb/cards/HALLMARK_IL6_JAK_STAT3_SIGNALING) | 1.56 | 0.008 | 0.026 |
| [HALLMARK_COMPLEMENT](http://www.broadinstitute.org/gsea/msigdb/cards/HALLMARK_COMPLEMENT) | 1.53 | <0.001 | 0.029 |
| [HALLMARK_APICAL_SURFACE](http://www.broadinstitute.org/gsea/msigdb/cards/HALLMARK_APICAL_SURFACE) | 1.5 | 0.034 | 0.029 |
| [HALLMARK_ANDROGEN_RESPONSE](http://www.broadinstitute.org/gsea/msigdb/cards/HALLMARK_ANDROGEN_RESPONSE) | 1.44 | 0.014 | 0.037 |
| [HALLMARK_ALLOGRAFT_REJECTION](http://www.broadinstitute.org/gsea/msigdb/cards/HALLMARK_ALLOGRAFT_REJECTION) | 1.33 | 0.031 | 0.076 |
| [HALLMARK_NOTCH_SIGNALING](http://www.broadinstitute.org/gsea/msigdb/cards/HALLMARK_NOTCH_SIGNALING) | 1.25 | 0.168 | 0.11 |
| [HALLMARK_GLYCOLYSIS](http://www.broadinstitute.org/gsea/msigdb/cards/HALLMARK_GLYCOLYSIS) | 1.24 | 0.038 | 0.111 |

Enriched gene sets in QGP-1/shLuc vs QGP-1/shPCK2 (down-regulated)

| GS | NES | p | q |
| --- | --- | --- | --- |
| [HALLMARK_UNFOLDED_PROTEIN_RESPONSE](http://www.broadinstitute.org/gsea/msigdb/cards/HALLMARK_UNFOLDED_PROTEIN_RESPONSE) | -2.14 | <0.001 | <0.001 |
| [HALLMARK_MTORC1_SIGNALING](http://www.broadinstitute.org/gsea/msigdb/cards/HALLMARK_MTORC1_SIGNALING) | -1.79 | <0.001 | 0.002 |
| [HALLMARK_MYC_TARGETS_V2](http://www.broadinstitute.org/gsea/msigdb/cards/HALLMARK_MYC_TARGETS_V2) | -1.75 | <0.001 | 0.003 |
| [HALLMARK_CHOLESTEROL_HOMEOSTASIS](http://www.broadinstitute.org/gsea/msigdb/cards/HALLMARK_CHOLESTEROL_HOMEOSTASIS) | -1.54 | 0.016 | 0.059 |
| [HALLMARK_MYC_TARGETS_V1](http://www.broadinstitute.org/gsea/msigdb/cards/HALLMARK_MYC_TARGETS_V1) | -1.42 | 0.001 | 0.167 |
| [HALLMARK_PI3K_AKT_MTOR_SIGNALING](http://www.broadinstitute.org/gsea/msigdb/cards/HALLMARK_PI3K_AKT_MTOR_SIGNALING) | -1.36 | 0.045 | 0.79 |
